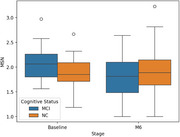# Changes in conversational pattern as a clinical trial outcome using the I‐CONECT data

**DOI:** 10.1002/alz70856_100936

**Published:** 2025-12-25

**Authors:** Liu Chen, Chao‐Yi Wu, Jiayu Zhou, Hiroko H Dodge

**Affiliations:** ^1^ Massachusetts General Hospital, Harvard Medical School, Boston, MA, USA; ^2^ University of Michigan, Ann Arbor, MI, USA

## Abstract

**Background:**

Linguistic measures derived from spontaneous conversations are behavioral indicators shown to be associated with cognitive functions. In this study, we investigated a natural language processing measure, semantic noise (SN), to quantify conversational patterns and monitor intervention‐induced changes. We hypothesized that (1) conversational patterns differ between older adults with mild cognitive impairment (MCI) and those with normal cognition (NC), and (2) after a high dosage of conversational interactions, the conversational patterns of individuals with MCI would resemble those of individuals with NC.

**Method:**

We analyzed transcriptions from semi‐structured conversations in the I‐CONECT project (ClinicalTrials.gov: NCT02871921). Participants engaged in 30‐minute semi‐structured conversations four times per week via online video chats for six months. Each video chat session followed a predefined topic based on the week and session index (e.g., dancing was the topic of the first conversation session in the second week). Fifty‐two participants (26 MCI and 26 NC) had at least one recorded video chat during the second week of the intervention (baseline for this study) and during the final week (i.e., 24th week) of the six‐month intervention. We measured semantic noise (SN) for each conversation session. The mean SN (MSN) from the second and 24th weeks was used to represent the baseline and six‐month (M6) values, respectively.

**Results:**

At baseline, the MCI group's MSN was significantly higher than the NC group's (MCI: 2.09 ± 0.34, NC: 1.87 ± 0.35; *p* =  0.024). After six months, no significant difference was observed between groups (MCI: 1.81 ± 0.47, NC: 1.95 ± 0.47; *p* =  0.280). In the MCI group, baseline MSN was significantly higher than M6 MSN (*p* = 0.009), but no significant change occurred in the NC group (*p* = 0.426).

**Conclusions:**

We demonstrated a conversational pattern difference between the MCI and NC groups at baseline. After six months of conversational interaction, the MCI group's MSN decreased, shifting from being significantly higher than that of the NC group at baseline to no significant difference at M6. This suggests that following the intervention, the speech pattern of the MCI group resembled that of the NC group.